# Isolated Piloleiomyoma Presenting as a Keloid Scar: A Case Report

**DOI:** 10.7759/cureus.88546

**Published:** 2025-07-22

**Authors:** Soukaina Lazouzi, Fouzia Hali, Bouchra Baghad, Soumiya Chiheb

**Affiliations:** 1 Department of Dermatology, University Hospital Center Ibn Rochd, Casablanca, MAR

**Keywords:** benign, keloid, leiomyoma, scar, tumor

## Abstract

Piloleiomyomas are uncommon benign tumors that originate from the arrector pili muscle and can resemble other skin lesions, such as keloids. We report the case of a 32-year-old female who presented with a solitary, painful, erythematous nodule on the extensor surface of the right arm, evolving over three years without preceding trauma. Initially misdiagnosed as a keloid and unresponsive to corticosteroid injections, the lesion was surgically excised, and histological and immunohistochemical analysis confirmed a piloleiomyoma. Postoperative recovery was favorable, with no recurrence at three months. This case highlights the diagnostic challenge posed by isolated piloleiomyomas and the importance of histological confirmation in atypical or treatment-resistant lesions resembling keloids.

## Introduction

Piloleiomyoma is a rare and benign smooth muscle tumor originating from the arrector pili muscle of the pilosebaceous unit (small muscles attached to hair follicles) [1]. It is the most common subtype of cutaneous leiomyomas, which themselves account for approximately 5% of all leiomyomas [2]. Their true prevalence remains unknown, partly because piloleiomyomas often share clinical features with other dermal nodules, such as firmness, tenderness, or pigmentation, leading to frequent misdiagnosis, particularly in isolated cases [2]. We report here the case of a patient who presented with a piloleiomyoma mistaken for a keloid scar.

## Case presentation

**Figure 1:**
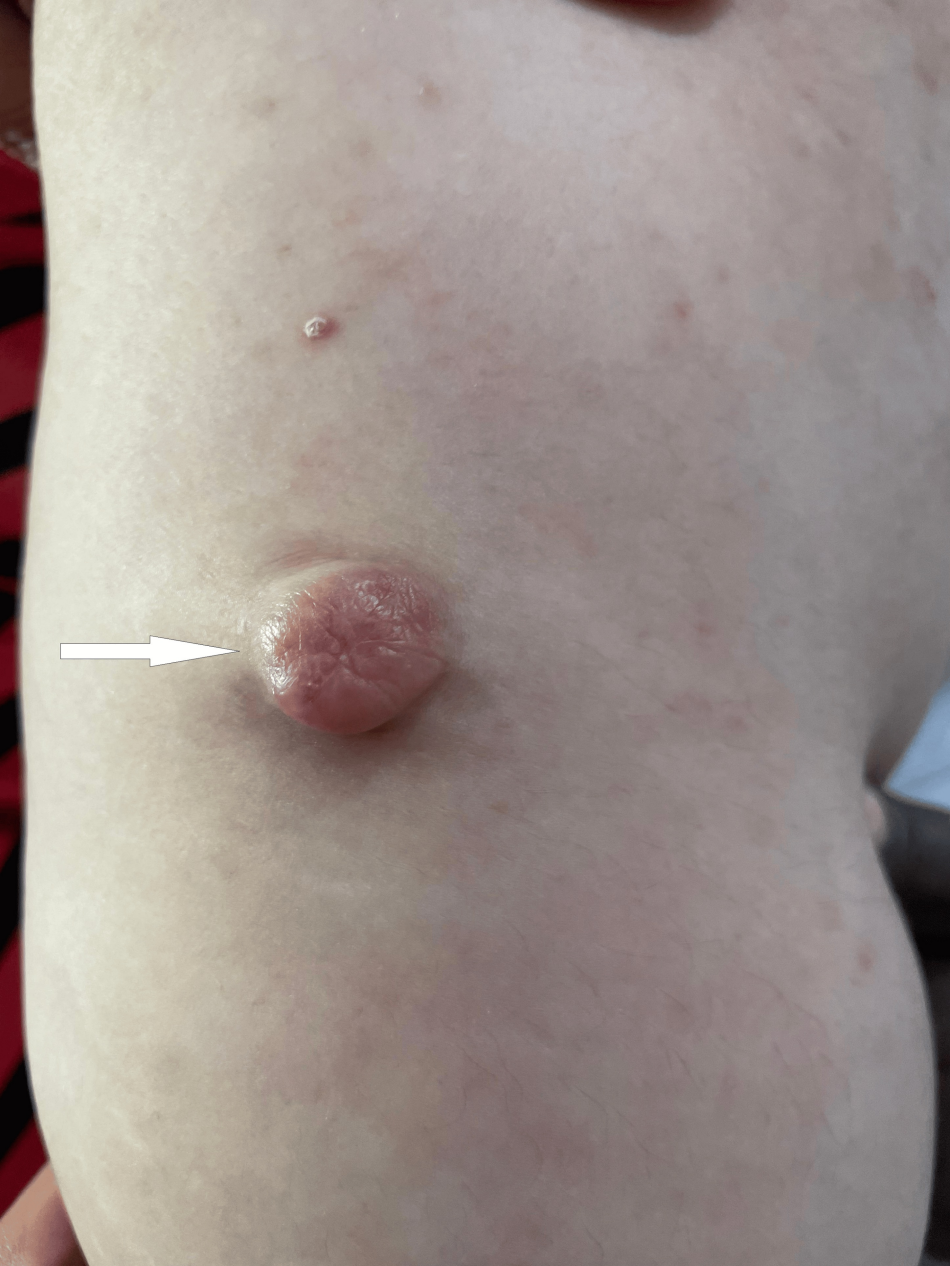
Solitary erythematous sessile nodule with a smooth, shiny surface and regular borders on the extensor surface of the right arm in a 32-year-old female patient, clinically resembling a keloid scar

A 32-year-old female patient with no previous personal or family history of similar skin lesions, tumors, or related genetic conditions presented with a painful lesion on the extensor surface of the right arm that had been evolving for three years. It initially appeared as an erythematous papule and gradually increased in size, with spontaneous intermittent pain and tenderness upon palpation and no history of trauma or other prior lesions. The patient had received three intralesional corticosteroid injections with no improvement. On examination, the patient was classified as Fitzpatrick phototype IV and presented with a sessile, dermal nodular lesion measuring approximately 2 cm in length. The lesion had a firm consistency, a smooth and regular surface, was erythematous with well-defined borders, and exhibited limited mobility at its base (Figure 1).

**Figure 2:**
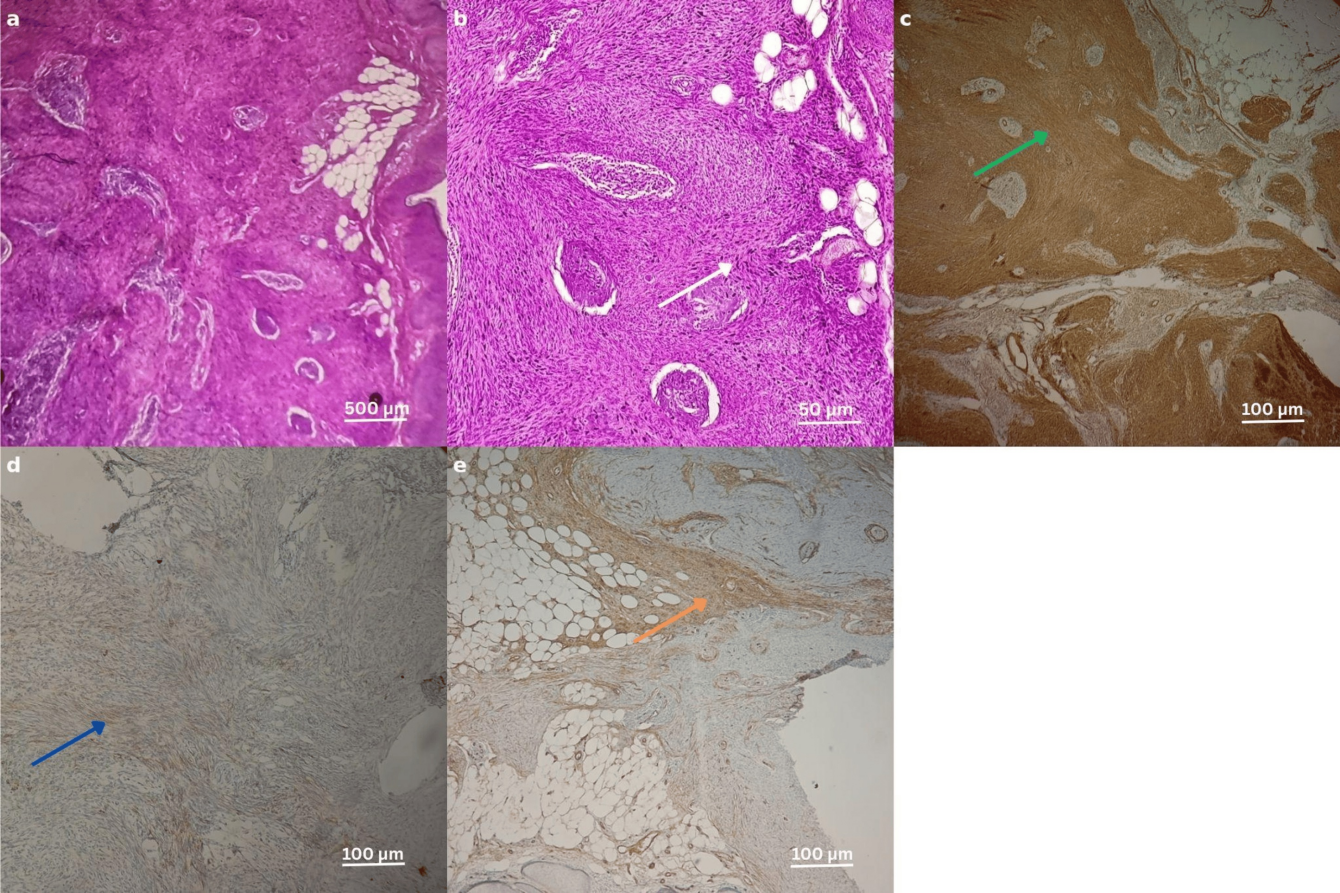
Histopathological and immunohistochemical features of the lesion (a) Low-power magnification (H&E stain, ×40) showing a dermohypodermal proliferation composed of interlacing fascicles of spindle cells. (b) High-power view (H&E stain, ×400) revealing spindle-shaped tumor cells with elongated, moderately atypical nuclei and visible mitotic figures (white arrow). (c) Strong and diffuse cytoplasmic expression of anti-smooth muscle actin in tumor cells (green arrow). (d) Focal cytoplasmic positivity for cytokeratin AE1/AE3 (blue arrow). (e) Heterogeneous and focal membranous expression of CD34 (orange arrow).

There were no other similar lesions or palpable lymphadenopathy. A surgical excision of the lesion with histological study revealed a dermohypodermal spindle cell tumor proliferation, expressing on immunohistochemical analysis anti-smooth muscle actin antibodies, desmin, and PS100 in a heterogeneous manner, focally CD34 and CK without expression of CD68 (Figure 2).

The subsequent outcome after three months was favorable, with no recurrence.

## Discussion

Cutaneous leiomyomas, also known as superficial leiomyomas, were first described by Virchow in 1854 and are estimated to account for five percent of all leiomyomas [2]. They include, depending on the original site, angioleiomyomas, genital leiomyomas, and piloleiomyomas, which are the most common variant [2,3].

Piloleiomyomas most often appear during the second or third decade of life, with no gender prevalence [4]. Typically affecting the extremities, followed by the trunk and rarely the head and neck, they most often present as tender or painful multiple, firm, skin-colored to light brown papules or nodules of up to 20 mm in diameter [2], with various distributions reported, such as symmetrical, blaschkoid, disseminated, and segmental or zosteriform [1-3].

Our case is distinct because of the solitary presentation mimicking a keloid and underlines the diagnostic difficulty in this type of situation, where differential diagnosis includes, along with spontaneous keloid eruption, dermatofibroma, cutaneous schwannoma, neurofibroma, eccrine spiradenoma, glomus tumor, and angiolipoma [2,5].

Histology of cutaneous leiomyomas shows an unencapsulated, well-defined dermal tumor made up of spindle-shaped cells with eosinophilic cytoplasm and elongated, cigar-shaped nuclei with halos [6]. While there may be some variation in nucleus shape and occasional mitoses, the tumor remains benign [4]. The presence of varying levels of epidermal hyperplasia and basal pigmentation, with trapped hair follicles and eccrine glands, is highly suggestive of piloleiomyoma [2].

Upon immunohistochemical examination, piloleiomyomas typically express smooth muscle markers such as actin, alpha-smooth muscle actin, calponin, and desmin, supporting their smooth muscle origin. Although uncommon, S-100 expression, observed in our patient, has been reported in approximately 20% of cases, as noted by Agulló Pérez et al. [7], and should be interpreted with caution given its association with nerve sheath tumors. Focal positivity for CD34 and cytokeratin AE1/AE3, also present in our case, is rare but has been documented in cutaneous leiomyomas.

Treatment is often necessary to relieve pain or sensitivity and involves smooth muscle relaxants such as nifedipine, phenoxybenzamine, nitroglycerin, and doxazosin alongside nerve-targeting drugs like gabapentin and topical analgesics: botulinum toxin injections and cryotherapy [2,6]. Surgical excision is the preferred approach for few or solitary and painful leiomyomas [5], though other methods, including electrodessication, radiotherapy, and carbon dioxide laser ablation, have been used with varying success [3,4].

Recurrence after surgery is possible [6], mainly in incomplete resection, but piloleiomyomas remain benign with a good prognosis [4]. In some cases, especially when multiple, they may be associated with hereditary conditions such as hereditary leiomyomatosis and renal cell cancer syndrome [6]. However, our patient had no clinical or familial features suggestive of such an association.

## Conclusions

Although uncommon, piloleiomyomas pose a significant diagnostic challenge due to their clinical resemblance to more common dermatologic entities such as keloids. This case underlines the importance of considering them in the differential diagnosis of persistent and painful dermal nodules, particularly when the lesion is solitary and unresponsive to standard therapies like corticosteroids. Accurate diagnosis relies on histological and immunohistochemical analysis. Surgical excision remains the treatment of choice for solitary lesions, while alternative approaches may be considered in multifocal or symptomatic cases.

## References

[1] Alper M, Parlak AH, Kavak A, Aksoy KA (2004). Bilateral multiple piloleiomyomas on the breast. Breast.

[2] Yan ZZ, Sun X, Zhang M, Chang JM, Gao YQ (2021). Piloleiomyoma. Int J Dermatol Venereol.

[3] Michajłowski I, Błażewicz I, Karpinsky G, Sobjanek M, Nowicki R (2015). Successful treatment of multiple cutaneous leiomyomas with carbon dioxide laser ablation. Postepy Dermatol Alergol.

[4] Machado BH, Tejada VF, Pitanguy I (2023). Cutaneous pilar leiomyoma: case report and analysis of therapeutic possibilities. Rev Bras Cir Plást.

[5] Jaganathan I, Kumar SD, Bhatnagar A, Mitra D, Bahuguna A, Mutreja D Cutaneous leiomyomas [IN PRESS]. Indian J Postgrad Dermatol.

[6] Bernett CN, Mammino JJ (2025). Cutaneous leiomyomas. StatPearls [Internet].

[7] Agulló Pérez AD, Resano Abárzuza MA, Córdoba Iturriagagoitia A, Aisa Rivera G, Patiño García A, Yanguas Bayona JI (2021). Cutaneous leiomyomas: a clinicpathological and epidemiological review [Article in Spanish]. An Sist Sanit Navar.

